# Multi-scale satellite observations of Arctic sea ice: new insight into the life cycle of the floe size distribution

**DOI:** 10.1098/rsta.2021.0259

**Published:** 2022-10-31

**Authors:** Byongjun Hwang, Yanan Wang

**Affiliations:** Department of Biological and Geographical Sciences, The University of Huddersfield, Queensgate, Huddersfield, HD1 3DH, UK

**Keywords:** Arctic sea ice, floe size distribution, power law, satellite, melt, wave

## Abstract

This study provides a new conceptional framework to understand the life cycle of the floe size distribution of Arctic sea ice and the associated processes. We derived the floe size distribution from selected multi-scale satellite imagery data acquired from different locations and times in the Arctic. Our study identifies three stages of the floe size evolution during summer – ‘fracturing’, ‘transition’ and ‘melt/wave fragmentation’. Fracturing defines the initial floe size distribution (*N* ∼ *d^−α^*, where *d* is floe size) formed from the spring breakup, characterized by the single power-law regime over *d* = 30–3000 m with *α* ≈ 2. The initial floe size distribution is then modified by various floe fragmentation processes during the transition period, which is characterized by ‘selective’ fragmentation of large floes (*d* > 200–300 m) with variable *α* = 2.5–3.5 depending on the degree of fragmentation. As ice melt intensifies, the melt fragmentation expands the single power-law regime into smaller floes (*d* = 70 m) with *α* = 2.4–3.8, while a significant reduction of small floes (*d* < 30–40 m) occurs due to lateral melt. The shape factor shows an overall progression from elongated floes into rounded floes. The effects of scaling and wave-fracture are also discussed.

This article is part of the theme issue 'Theory, modelling and observations of marginal ice zone dynamics: multidisciplinary perspectives and outlooks'.

## 1. Introduction

Arctic sea ice undergoes a seasonal life cycle from spring breakup to summer melt out. During the spring breakup, the continuous ice cover is fragmented into discrete floes of different sizes [1]. The size and geometric characteristics of those floes are important in understanding ice dynamics (i.e. form drag, [2]), ice thermodynamics such as lateral melting [3,4], ice rheology [5] and air–ice–ocean momentum exchange [4,6–9].

The floe size-related processes are much more active in the marginal ice zone (MIZ), defined as an ice-covered region where waves and swell penetrate from open water [10] or an ice-covered area with sea ice concentration of 0.15–0.80 [11–13]. As the MIZ in the Arctic has become a dominant feature in the Arctic [11,13] or will even intensify in the future [12], understanding the floe size distribution and the associated characteristics and processes is increasingly important for sea ice/climate modelling (e.g. [14,15]).

The primary objectives of this paper are to (a) provide an overview of the progress and issues within the observations of the floe size distribution and (b) propose a concept of the life cycle of the floe size distribution with examples from the observations. As reviewed below, the observations of the floe size distribution in the Arctic have been rather inconsistent and incomplete, e.g. the floe size distribution has been observed, measured and presented differently among researchers, although there has been an effort to reconcile the inconsistency among the observations [16]. The conceptional work proposed by this study may form a starting point for harmonizing existing datasets from a wide range of satellite and aerial observations and pointing to better coordination of future observations to inform the seasonal cycle of the floe size distribution and an insight into the associated processes.

## 2. Previous observation of the floe size distribution

Previously, various satellite or airborne observations have been used to measure the floe size distribution and understand its behaviour in different seasons and locations in the Arctic. The very first observation of the floe size distribution in the record was done by Vinje [17] who used Landsat imagery to study the dynamic behaviour of large floes (area greater than 10 km^2^) in the Fram Strait. In 1980, Weeks *et al*. [18] analysed side-looking airborne radar imagery with the image resolution or pixel spacing (*δ*) of approximately 25 m to study the geometry and size of multi-year ice floes (floe size range of *d* = 100–3600 m) in the Beaufort Sea. A systemic study of the floe size distribution was conducted by Rothrock & Thorndike [19]. They analysed aerial photographs and Landsat imagery to derive the floe size and geometric properties ranging between 100 and 20 000 m.

Since then, the observations carried out at various scales derived the floe size distribution over a wide range of floe sizes from 1 up to 20 000 m (electronic supplementary material, table S1). Aerial photograph mosaic (*δ* ∼ a few centimetres) can provide the floe size distribution in the range of *d* = 1–5 to 50–100 m [20,21]. On the other hand, Landsat (*δ* = 30 m), ERS-1 SAR (*δ* = 100 m) and MODIS (*δ* = 250 m) images can provide the floe size range of *d* = 100–1000 m [21], 900–10 000 m [22] and 2000–20 000 m [23], respectively. Previous studies (see electronic supplementary material, table S1) used high-resolution visible images (*δ* = 1 m) from the USGS Global Fiducials Library (GFL) (http://gfl.usgs.gov/), declassified by the MEDEA group (hereafter MEDEA) [24]; others used TerraSAR-X/TanDEM-X (hereafter TX) StripMap (SM) images (*δ* = 2.75 m). The floe size distribution derived from MEDEA images typically covers the floe size ranges of *d* = 5–10 to 1000 m [23,25,26], while TX SM images cover *d* = 200 to 3000 m [1].

The floe size distribution in the Arctic has been characterized by a single power law [1,19,23,25], two power-law regimes [21,27] or different functions [28–30]. Previous observations widely report a single power-law behaviour, *N* ∼ *d^−α^*, where *N* is the non-cumulative floe size distribution, *d* is the mean calliper diameter of the floes and *α* is the exponent (e.g. [1,19,23]). The single power-law *α* ranges from 1.6 to 3.6 in the Arctic (expressed in non-cumulative form, some studies reported the exponents in cumulative, which were converted to non-cumulative form by adding one – see [16]). Some observations reported that the floe size distribution exhibits two power-law regimes with the transition point at *d* = 40 m [21] or 200–850 m [27]. Two power-law regimes are still in debate as (a) they may result from the finite image size in cumulative distribution [16], (b) power-law fits over a narrow range of the floe sizes are highly uncertain, (c) some processes (e.g. ice breaking by waves) can have a normal distribution peaked at preferred floe sizes [28,30], or (d) two power-law regimes may appear or not depending on the choice of threshold for separating ice from water in the images [26].

Previous studies have observed the seasonal evolution of the floe size distribution from spring through summer [1,23,26,31]. These studies show that large floes break into smaller floes during the spring-to-summer transition, i.e. a steeper slope in the floe size distribution (i.e. an increase in *α*). In late summer, melt out of small floes reduces the number of small floes [31]. During the freeze-up, the slope tends to be shallow (i.e. decrease in *α*) due to refreezing of leads and floe welding [23,26,31]. Process-based evolution of the floe size distribution has been the focus of previous observational studies. The floe fragmentation by wind, current and waves has been widely observed [22,25,30–33]. Both observations and modelling studies highlight the importance of waves/swell in floe fragmentation [31,34–36]. However, recent studies highlighted that thermodynamic ice melt can play a significant role in floe fragmentation [1,33].

Previous observations have been conducted to examine changes in the floe size distribution during storm or wave events [22,25], following specific drifting sites [1,31], or in a region to examine seasonal cycle [23] or scaling behaviour [21]. For example, *α* varies from 3.0 to 3.2 (in non-cumulative form) at the SHEBA drifting site in 1998 [31], while it varies from 2.5 to 5.0 at the ONR MIZ drifting site in 2014 [1] or from 2.0 to 2.8 in the Beaufort and Chukchi Seas in 2013–2014 [23]. These variable floe size characteristics observed from those studies are mainly due to a wide range of variabilities in the atmospheric and oceanic forcing operating at the drifting sites or in regions as well as different ice conditions and seasonal/interannual variabilities. The observations of the floe size distribution are still scarce to capture the full range of the variabilities within the Arctic, especially for small floes or across different scales. In this study, we argue that a conceptional framework is a way to digest the observations at different scales and times/locations to improve our understanding of the characteristics of the floe size distribution and the associated fragmentation and melt processes, which can inform modelling efforts more effectively.

## 3. Hypothesis

During the spring breakup, the continuous ice cover breaks into discrete ice floes (hereafter we call it ‘fracturing’), which forms the initial floe size distribution. This initial floe size distribution may have a single power-law behaviour with the exponent (*α* ≈ 2) [37], which can be strongly related to sea ice fracturing and deformation that have multi-fractal and scale-invariant behaviours [38,39]. Before the ice melt intensifies, large ice floes break into smaller ones by floe-floe interactions, a flexural failure by ocean surface waves/swells [36,40–42], deforming failure due to wind or ocean currents [33,43], or swells travelling a long distance [44] (hereafter we call this period ‘transition’). As the ice melting intensifies, thinner ice or melt ponds within the floes act as ‘weak’ points enhancing the floe fragmentation into smaller floes [1,33,43]. Increased open water areas generate stronger ocean surface waves [45] that further fragment weakened ice floes into smaller floes [36,40–42] and the lateral melt intensifies among small floes [3,46] (hereafter we call this period ‘melt/wave fragmentation’). The final stage would be a complete melt out of the floes in the presence of ice bands [47,48]. In this study, we hypothesize that the seasonal life cycle of the floe size distribution can be conceptualized in three stages: fracturing, transition and melt/wave fragmentation and each stage of the life cycle has unique floe size distribution characteristics across scales.

## 4. Floe size evolution and properties

### (a) Description of data and methods

To address the hypothesis, five different types (and resolutions) of satellite imagery data were used in this study (see electronic supplementary material, table S2 for detailed information on the data used). Worldview-3 (WV3) imagery provides the highest resolution (*δ* = 0.3 m), and MEDEA and TX Spotlight (SL) images provide the resolution of *δ* = 1.00 and 1.25 m, respectively, while TX SM and ScanSAR (SC) images provide the resolution of *δ* = 2.75 and 8.25 m, respectively. The MEDEA images were acquired either at the fixed GFL sites (http://gfl.usgs.gov/) or following the ONR MIZ drifting site [49]. Two GFL sites are in the Chukchi Sea (70° N and 170° W) (hereafter ‘chukchi’) and in the Fram Strait (84.9° N and 0.5° E) (hereafter ‘fram’). At the MIZ drifting site, we used the MEDEA images acquired at Cluster 2 and Cluster 4 (i.e. ‘miz02’ and ‘miz04’, see [49]). Two TX SL images were acquired at the MOSAiC drifting site [50]. The WV3 image was acquired in the Bering Sea to represent the floe size distribution in the presence of waves. The floe size distribution was derived from these images using the algorithm described in Hwang *et al*. [51]. The floe size (*d*) is measured as the mean calliper diameter (in metres) of a floe, and the shape factor is the ratio of the major axis to the minor axis of an ellipsoid fitted to individual floes [37]. In this study, the floe size distribution is presented in a non-cumulative distribution, *N ∼ d^−α^*, following Stern *et al*. [16]. For the power-law fit estimate of the floe size distribution, the maximum-likelihood estimator (MLE) [52,53] was used in this study (the same estimator has been used in previous studies [1,23,26] and refers to those studies and electronic supplementary material, table S2 for further information).

### (b) Fracturing

**Figure 1.**
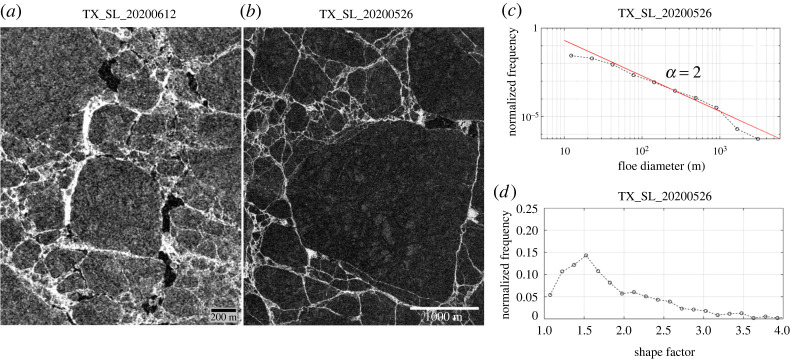
TerraSAR-X/TanDEM-X (TX) Spotlight (SL) imagery showing fracturing of the ice. Two TX SL images were acquired on (*a*) 12 June 2020 and (*b*) 26 May 2020 at the MOSAiC drifting site. The 26 May 2020 TX SL image was analysed to derive (*c*) the floe size distribution and (*d*) shape factor. In the image, the ice floes are in a dark tone and the fracture lines are in a bright tone due to the backscattering inversion (see text). (Online version in colour.)

Fracturing defines the initial size distribution of the discrete floes, resulting from the spring breakup (i.e. a continuous ice cover to discrete floes). During the spring breakup, fracturing is associated with cracks/leads and fracture lines created by deformation events and the strength of the refrozen leads/cracks is highly related to the timing of the events (i.e. newer leads/cracks are more vulnerable to fracture). During the spring melt, the fracture lines are often shown bright (high backscattering) in the SAR imagery due to increased roughness from brash ice within the leads/cracks [54]. These fracture lines are effective in breaking off the continuous ice into discrete floes. This can be seen in the TX SL image acquired on 26 May 2020 and 12 June 2020, showing the floes are being detached along the bright-toned fracture lines (figure 1*a*). As discrete floes would be formed mainly through the fracture lines, we can consider the fracture pattern as the determining factor for the initial floe size distribution. To test this assumption, we analysed the TX SL image acquired on 26 May 2020 (figure 1*b*) and derived the floe size distribution and shape factors from the image. The derived floe size distribution follows closely with a single power-law with *α* ≈ 2, although the confidence of being the power-law regime is low (*p* < 0.1, see electronic supplementary material, table S2) and some deviations from the power-law fit are observed for the floes larger than *d* = 1000 m or smaller than *d* = 50 m (figure 1*c*). The peak of the shape factor distribution occurs at 1.5 (figure 1*d*), which is close to the reported value in [37].

**Figure 2.**
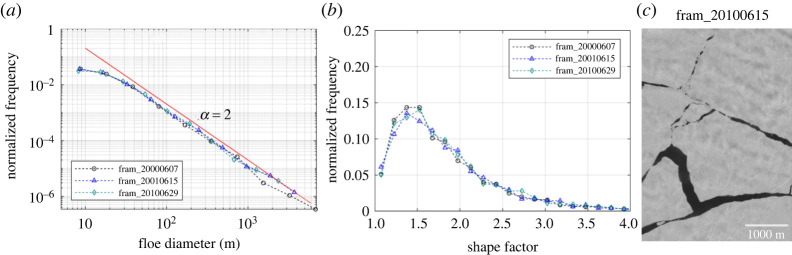
(*a*) Normalized frequency of floe size distribution and (*b*) normalized frequency of the shape factor derived from MEDEA images during fracturing. A sub-section of the MEDEA images acquired on 15 June 2010 in the Fram Strait is shown in (*c*). (Online version in colour.)

To further examine the power-law behaviour of the initial floe size distribution resulting from fracturing, we analysed three MEDEA images acquired at the GFL sites in the Fram Strait between 2000 and 2010. The selected images all show that the floes are freshly broken off with small floes in between (electronic supplementary material, figure S2), which represents the initial state of the floe size distribution right after the spring breakup, yet with different atmospheric and oceanic conditions leading to the initial floe size distribution (as acquired in different years). The results show a remarkable resemblance between the derived floe size distributions (figure 2*a*). The floe size distributions all follow a single power law of *α* = 2 for the range of *d* = 30 to 3000 m (figure 2*a*) (see electronic supplementary material, table S2 – the estimated *α* = 1.99 to 2.07 with high confidence (*p* > 0.1) and electronic supplementary material, figure S1 - the number histogram plots also showing a typical power-law pattern). The distributions of the shape factor distributions show a peak at 1.4–1.5 and the number densities are smaller than 0.15 (figure 2*b*).

Gherardi & Lagomarsino [37] reported the peak of the shape factor distribution occurred at 1.5 or slightly larger (fig. 4 in their paper), and their results show the same power-law behaviour with *α* = 2. Note that they used multiple satellite images (*δ* = 2–30 m) that represent spring conditions (i.e. June in the Barents Sea and Svalbard area; March in the Kara Sea), which is similar to the floe condition in our cases. The floe size distributions for the floes smaller than *d* = 30 m deviate from the power-law (flattening). This can be attributed to the limitation of the image resolution. In the analysis, we exclude the floes that contain less than 25 pixels to avoid erroneous results caused by internal image variation or noises. Therefore, the minimum retrievable floe size would be 5–10 m (depending on floe shape) for MEDEA images. Flattening in non-cumulative distribution was observed in other studies [16,37], which is typically excluded from the discussion.

### (c) Transition

In the previous section, we examined the power-law behaviour (in non-cumulative distribution) and shape factor of the floes resulting from the spring breakup (fracturing). We now investigate a transitional period from fracturing (i.e. the initial floe size distribution) leading to summer melt. To examine this, we select six MEDEA images (four from the Chukchi Sea site and two from the Fram Strait site), displaying floe conditions of some progression from the initial fracturing stage. The selected images show more dynamic ice floe conditions than the fracturing cases, i.e. floes look more rounded and separated from one another (electronic supplementary material, figure S3). We have no information on how long ago the initial fracturing had occurred, however, the selected images appear to be not freshly broken off into discrete floes, yet a network of melt ponds has not been well developed (electronic supplementary material, figure S4). We speculate that those floes have gone through dynamic processes such as floe-to-floe interactions, and wind- or swell/wave-induced floe failures.

**Figure 3.**
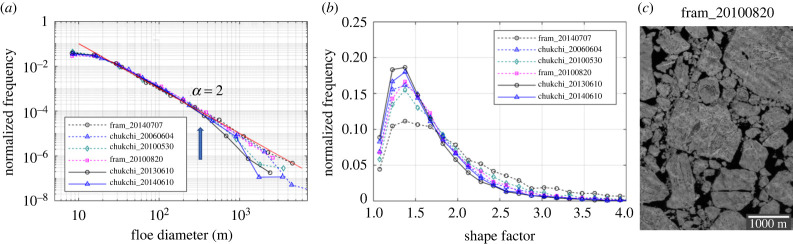
(*a*) Normalized frequency of floe size distribution and (*b*) normalized frequency of the shape factor derived from MEDEA images during transition. A sub-section of the MEDEA image acquired on 20 August 2010 in the Fram Strait is shown in (*c*). (Online version in colour.)

The floe size distributions derived from those images tend to follow the same single power law with *α* = 2 for the floe size range of *d* = 30 to 200–300 m (figure 3*a*), although the power-law fit fails over the short floe size range (see electronic supplementary material, table S2). The peaks of the shape factor distributions are slightly shifted to a smaller number (1.35–1.4) than the fracturing cases and the number densities for the shape factors increase above 0.15 (figure 3*b*).

The floe size distributions derived from those images show downward curvatures after *d* = 200–300 m (see the blue arrow in figure 3). Such concave-down curvatures at large floes were observed in other studies and were attributed to the under-sampling of the largest floes in the limited image size [1,23]. However, the floe size distributions from the fracturing cases do not show such downward curvatures at the largest floes (figure 2*a*), and the sizes of the images for the transition cases are not particularly smaller than those of the fracturing cases (see electronic supplementary material, table S2). For example, the image dimension of the fram_20100629 image (fracturing) is 10 608 by 5040 m, while the image dimension of the chukchi_20060604 image (transition) is 13 486 by 17 557 m. This indicates that the finite image size may not be the major cause for the downward curvatures at the largest floes for the transitional cases.

To obtain an insight into the different behaviour at large floes for the transition cases, we investigated the power-law behaviour for the floes larger than 200–300 m. The MLE results show that a significant (*p* > 0.1) power-law regime exists for the large floes (approx. 200–300 m < *d* < 3000 m) with a steeper slope (*α* = 2.54 to 3.46) (see electronic supplementary material, table S2). This suggests that a selective floe fragmentation may occur for large floes (greater than 200–300 m), while small floes (*d* < 200–300 m) are not affected by fragmentation and maintain the initial floe size distribution from the spring breakup.

### (d) Melt/wave fragmentation

In the previous section, we argued that the floe size distribution during the transition period is characterized by a power law of the extreme tail of the distribution for large floes (greater than 200–300 m), indicating selective floe fragmentation of large floes from the initial floe size distribution (i.e. fracturing). In this section, we will examine the characteristics of the floe size distribution for the floes experiencing significant thermodynamic ice melt. To examine this, we selected three MEDEA images that were acquired at the MIZ drifting site in 2014. These images are unique as they follow the drifting site where different types of autonomous buoys were deployed to measure the thermodynamic and dynamic variables of ice, ocean and atmosphere [1,55]. The three images were taken on 18 July, 30 July and 14 August 2014. The collocated ice mass balance buoys indicate considerable surface and basal ablation of the ice during this period (fig. 14 in [1]).

**Figure 4.**
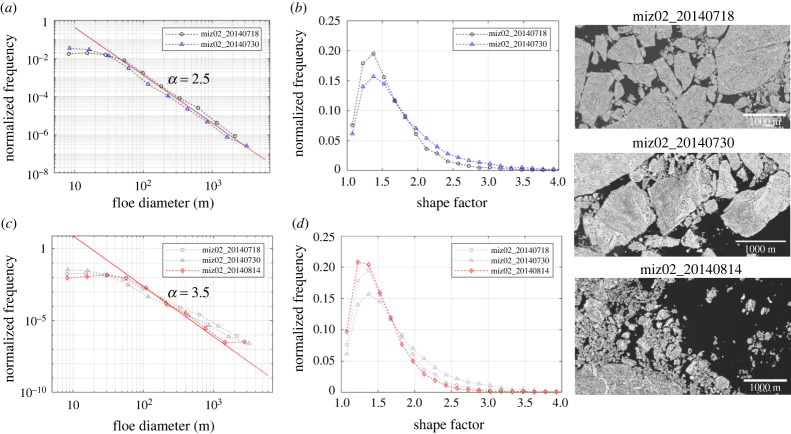
(*a*) Normalized frequency of floe size distribution and (*b*) normalized frequency of the shape factor derived from MEDEA images during early melt, and the corresponding (*c*) floe size distribution and (*d*) shape factor during late melt. The images on the right panel show sub-sections of the MEDEA images acquired during early and late melt. (Online version in colour.)

The floe size distributions derived from the three images no longer show any downward curvatures at the largest floes, but instead, follow a single power law with *α* = 2.5 for the two July images (figure 4*a*) and *α* = ∼3.5 for the August image for *d* = 70 to 2000 m (figure 4*c* and electronic supplementary material, table S2). All cases show steeper slopes than the ones from the fracturing (*α* = 2.0), and the range of *α* and the distribution of shape factors are comparable with the transition cases (electronic supplementary material, table S2). The peaks of the shape factor distribution occur at 1.35, similar to those from the transition cases (figure 3*b*), but slightly smaller than the ones for the fracturing cases (figure 2*b*). Another point to note is that the floe size range for the power-law fits are different between the images. For the 18 July image and 30 July image, the power law fits for the floe size range of *d* = ∼70 to 2000 m (figure 4*a* and electronic supplementary material, table S2), while for the 14 August case, the power law fits over the narrower floe size range of *d* = 70–1000 m (figure 4*c* and electronic supplementary material, table S2). Although the range of *α* and the shape factor distribution are comparable between transition and melt/wave fragmentation cases, the images show clear differences between the transition and melt/wave cases. For the melt/wave cases, a network of melt ponds is well developed, while the surface melt is still in progress for the transition cases (electronic supplementary material, figure S4). This indicates that the floe size distributions for the transition cases are unlikely caused by ‘melt’ fragmentation of heavily melted floes like we see in the melt/wave cases.

The floe conditions reflect the significant changes in the power-law behaviour on 14 August. The most noticeable change is a sudden increase of small, fragmented floes (less than a few hundred metres) on 14 August, compared with those on 18 and 30 July (see electronic supplementary material, figure S5). These changes suggest that there is a time-variant process (or processes) that causes the floe fragmentation. Hwang *et al.* [1] attributed this to thermodynamic melt that reduces the thickness of thinner ice to the critical point of the disintegration of the floes. Wang *et al.* [25] analysed the floe size distribution from the MEDEA images at 74° N (close to the location of our 14 August image) and found changes in the floe size distribution (decrease in number for the floes larger than 10 m) between 2 and 9 August images. They speculated that the change in the floe size distribution might be the result of melting and wave fracture, yet it was inconclusive how significant wave fracture was for the floe fragmentation. In addition, the 14 August image was taken 170 km from the ice edge, and the on-ice wave buoys installed on the floes did not record any wave actions that were strong enough to cause the bending of the floes at the image location [1].

**Figure 5.**
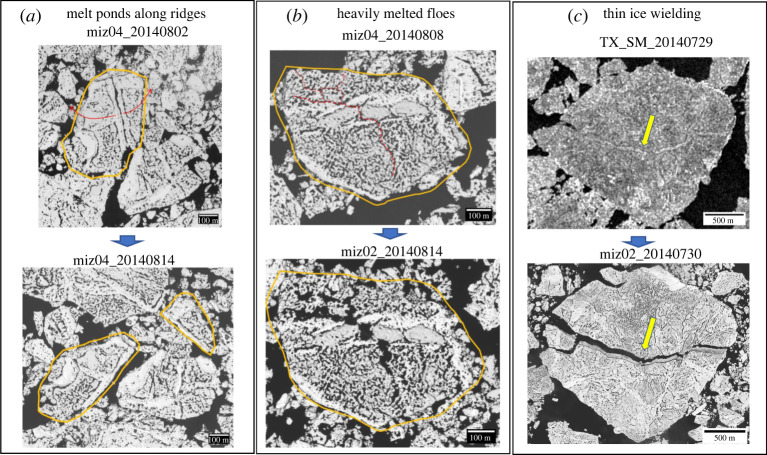
Close-up images showing floe fragmentation by melt (see text). In image (*a*) the yellow outlines mark the floe boundary before and after the fragmentation and red arrows mark the breaking up of the floe along the deepened melt ponds and ridges. In image (*b*), the yellow solid outlines mark the floe boundary and red dotted lines mark the fracture lines along the network of melt ponds. In image (*c*) yellow arrows mark the thin ice breakage. (Online version in colour.)

Although it is still debatable whether thermodynamic melt or waves is the dominant cause of the fragmentation, there is good evidence of ‘melt fragmentation’ from the images. Figure 5 shows examples of melt fragmentation in action. The 2 August image at the MIZ drifting site (Cluster 4) shows an ice floe with two lines of the ridges existing vertically and a long line of depression (valley) that contains deepened melt ponds between the ridges (the upper panel in figure 5*a*). The image acquired 13 days later shows the ice floe breaking apart into two floes along the ‘valley’ between the ridges (the lower panel in figure 5*a*). From the close examination of the images, we found melt ponds are well developed along the ridges, which is also observed by Arntsen *et al*. [33]. These melt ponds eventually act as weak points for the floe to break. A similar situation can occur in heavily melted ice floes. For example, the floe on 14 August at the MIZ drifting site (Cluster 2) looks like the surface melt was well underway, and the fragmentation occurred along with the network of melt ponds for 6 days (figure 5*b*). The case on 30 July is different. The fragmentation, in this case, occurred along the thinner ice that likely reached the critical thickness (figure 5*c*). These observations indicate that there are at least three different melt fragmentation mechanisms: (a) breaking off along the deepened melt ponds beside the ridges, (b) disintegration of heavily melted floes by the network of melt ponds and (c) thin-ice breakage welding thicker ice. Further studies will be required to gain a better understanding of melt fragmentation processes. For the effects of waves on fragmentation, refer to the discussion in §5.

## 5. Discussion and conclusion

### (a) Scaling

**Figure 6.**
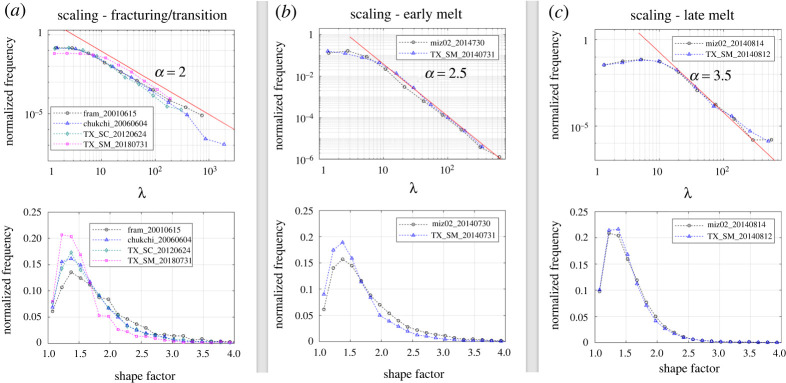
Comparison of floe size distribution and shape factor derived from the images with different resolutions. In graph (*a*), the floe size distribution and shape factor from the MEDEA (*δ* = 1 m) images are compared with those from TX SC (*δ* = 8.25 m) and TX SM (*δ* = 2.75 m) images. In graphs (*b*) and (*c*), the floe size distribution and shape factor from the MEDEA (*δ* = 1 m) images are compared with those from the TX SM (*δ* = 2.75 m) images. The cut-off diameters (*d*_c_) to calculate *λ* are 5 m for MEDEA, 50 m for TX SC and 15 m for TX SM. (Online version in colour.)

In previous sections, we found that the floe size distribution (non-cumulative distribution) follows a single power-law behaviour during fracturing, transition and melt/wave fragmentation. In this section, we examine whether the characteristics of the floe size distribution from MEDEA images can be comparable with those derived from lower resolution images (i.e. TX SM and TX SC). For this purpose, we selected four TX images—two images representing the transition cases and two images representing early and late melt cases. The floe size distributions and shape factors from the TX images are compared with those from MEDEA images in figure 6. For the comparison, the floe size was rescaled to *λ* (= *d*/*d_c_*), where *d_c_* is the cut-off diameter. *d_c_* is selected based on the minimum floe size and manually adjusted (in our case, *d_c_ *= 5 m for MEDEA, *d_c_ *= 15 m for TX SM, *d_c_ *= 50 m for TX SC and *d_c_ *= 1.9 m for WV3) [37].

For the transition cases, we selected a TX SC image (acquired on 24 June 2012 in the Chukchi Sea) and a TX SM image (acquired on 31 July 2018 in the Fram Strait). Based on the appearance of ice floes and dates/locations, the two TX images represent the floe conditions during transition rather than during fracturing (see electronic supplementary material, figure S6a,b). This is also partly supported by the fact that the shape factors from the two TX images have the floe size distributions close to the transition cases (figure 6*a*). It should be noted that TX images taken right after the spring breakup are often difficult to delineate the floe boundary and derive the floe size, as TX images contain speckle noises and have lower resolutions than MEDEA images.

The comparison of the floe size distribution (in *λ*) shows that the power-law regimes of the two TX cases have steeper slopes than the fracturing cases (figure 6*a*). The power-law exponent *α* from the TX SC image (*δ* = 8.25 m) is 2.44 over the floe size range of *d* = 300–10 000 m, *α* from the TX SM image (*δ* = 2.75 m) is 2.66 over the floe size range of *d* = 405–3500 m (electronic supplementary material, table S2). Both cases show high confidence (*p* > 0.1) in being a power-law regime. The low-cut limit (i.e. the smallest floe size of the power-law regime) from the TX SM image is larger than that from the TX SC image, although the resolution of the TX SC image is much lower. Despite this, the overall range of the low-cut limits from the TX images are comparable with those from the MEDEA images for the transition cases.

For the melt/wave fragmentation, we selected two TX SM images to compare the corresponding MEDEA images. In this case, the selected TX SM images were taken at the same drifting site (i.e. cluster 2 of the MIZ drifting site) [55]. The comparison results show an overall good match between the two scales, although the exponent *α* derived from the TX SM images (*α* = 2.8 to 3.8) is slightly higher than those from the MEDEA images (*α* = 2.5 to 3.5) (figure 6*b,c* and electronic supplementary material, table S2). The distributions of the shape factor are closely matched between the two scales, especially for the late melt case (figure 6*c*), although the normalized frequency from the TX SM image is slightly larger than the one from the corresponding MEDEA image for early melt (figure 6*b*). For the MEDEA images, the power-law fits cover the floe size ranges of *d* = 69–2000 m on 30 July (figure 4*a* and electronic supplementary material, table S2) and *d* = 70–1000 m on 14 August (figure 4*c* and electronic supplementary material, table S2). The power-law fits from the TX SM images (*δ* = 2.75 m) cover the floe ranges of *d* = 204–6000 for the early melt case and *d* = 298–2000 m for the late melt case (electronic supplementary material, table S2). The higher low-cut limits from the TX SM images are likely due to the inability to resolve small floes in the TX SM images.

### (b) Melt or waves?

The wave-induced breakup has been long considered an important physical process determining the floe size distribution from observations [22,25,30,32,56] and modelling studies [36,41,57]. However, recent studies [1,33] and our findings (in §4) suggested that melt fragmentation can play a very significant role in floe fragmentation, especially during late summer conditions. To examine the importance and characteristics between melt and wave-induced fragmentation, we analysed a WV3 image acquired on 28 April 2021 in the Bering Sea to represent the floe size distribution in the presence of significant wave actions. The floe size distribution derived from the WV3 image was then compared with the ones from MEDEA images acquired on 14 August (the case for melt fragmentation in §4).

**Figure 7.**
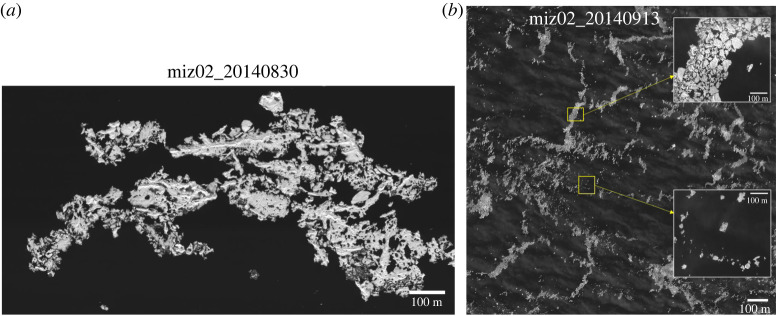
(*a*) A close-up image of an ice floe on 30 August 2014 and (*b*) a sub-section of the 13 September 2014 MEDEA image.

For the 14 August 2014 case (figure 6*c*), it is inconclusive whether waves also played a significant role in floe fragmentation. To investigate this matter further, we followed the aftermath of the 14 August fragmentation. Figure 7*a* shows the close-up image of a heavily melted floe on 30 August 2014 at the MIZ drifting site. From a careful examination of the floe, we can confirm that it is a single floe of approximately 1 km long, not an amalgamation of discrete floes, although some of the connections appear to be very fragile. We argue that such weak connections would have broken off if there were any moderate wave actions at the site.

However, the image acquired on 13 September 2014 following the same MIZ drifting site shows a quite different picture. It shows the multiple ice bands, i.e. typical features at the ice edge caused by wind waves [47] (figure 7*b*). Ice band is a composition of small floes (see the upper insert in figure 7*b*), likely fragments of heavily melted floes seen on 30 August (figure 7*a*). Estimating the floe size distribution at this stage is very difficult as small floes tend to be clustered together. From close-up images for the areas with dispersed floes, we can estimate that most of the floes are smaller than 100 m (see the lower insert in figure 7*b*). Given such small floe sizes, lateral melt increasingly becomes a significant factor especially for small floes (*d* < ∼30–50 m) [3], or even accelerates as open water areas quickly increase due to ice banding and subsequent solar heating [58], which leads to melt out of floes into open water.

**Figure 8.**
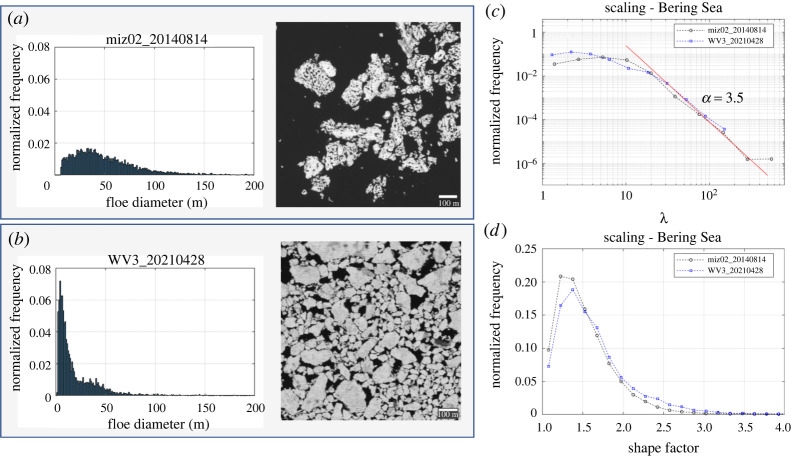
Floe number histograms from (*a*) the 14 August 2014 MEDEA image and (*b*) the 28 April 2021 WV3 image. Floe size distribution and shape factor between the MEDEA and the WV3 images are shown in (*c*) and (*d*), respectively. The cut-off diameters (*d*_c_) to calculate *λ* are 5 m for MEDEA and 1.9 m for WV3. WV3 2021 Maxar Technologies. (Online version in colour.)

In figure 8, we compare the floe size distributions between the WV3 image (the Bering Sea - waves) and the MEDEA image (Beaufort Sea - melt). By comparing two images at the same scale, we can see some differences in how the floes look like. The floes in the MEDEA image look heavily melted and porous (showing mature melt ponds and melt-out ridges) (figure 8*a*), while the floes in the WV3 image have no melt ponds on the surface (figure 8*b*). The corresponding number histograms show different characteristics. The number histogram from the MEDEA image shows a peak at approximately 30–40 m (figure 8*a*), while the number histogram from the WV3 image shows a combination of a tapered power law and a normal distribution (figure 8*b*) that is remarkably similar to the floe size distribution from the laboratory wave experiments in Herman *et al*. [30]. The reduction of small floes (*d* < 30–40 m) in the MEDEA image (figure 8*a*) suggests melt-out of small floes due to lateral melt. Hall & Rothrock [59] estimated a mean lateral melt rate of up to 0.1 m per day from sequential aerial photographs. At this melt rate, it would take approximately 100 days to completely melt out a 20-m floe (assuming a circle). At the MIZ drifting site, ice melt started on 1 July [1], so it is almost 45 days of melt until 14 August. This implies that an almost two times higher lateral melt rate would require melting out small floes (*d* ≈ 20 m). It should be noted that the 0.1-m lateral melt rate was observed by Hall and Rothrock during mid-July 1984 in the Greenland Sea [59]. Since then, *in situ* observations of lateral melt are scarcely limited. Considering the post-2000 observation of intensive melt due to solar warming [58], we may expect a higher lateral melt rate with declining Arctic sea ice than what Hall and Rothrock observed in 1984.

**Figure 9.**
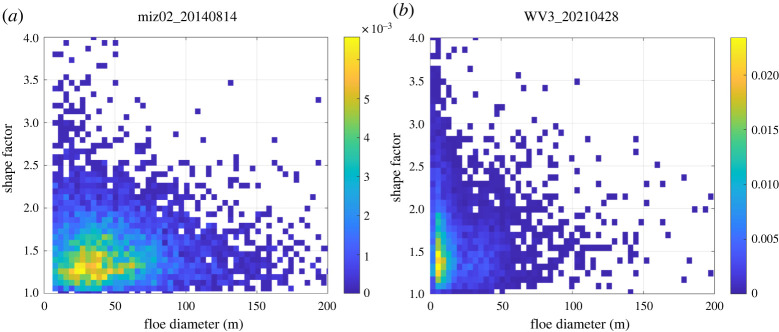
Probability density of points in the dimension of floe diameter and shape factor. (*a*) MEDEA-derived data (miz02_20140814) highlighting thermodynamic melt and (*b*) WV3-derived data (WV3_20210428) highlighting wave-fracture. (Online version in colour.)

In terms of power-law behaviour and shape factor, both cases show a single power-law behaviour, although the floe size range fitted to the power law is shorter for the WV3 (Bering Sea) case (*d* = 39 to 400 m (figure 8*c* and electronic supplementary material, table S2). Herman *et al.* [30] argued that a shorter floe size range is one of the reasons against whether a power law really exists, yet it is interesting both cases are fitted to the power law with the similar exponents (*α* = 3.29–3.45). For the shape factor, the peak for the WV3 case is slightly larger (=1.33) than the one for the MEDEA image (=1.22) (figure 8*d*), indicating more elongated floes for the Bering Sea case. For the MEDEA image, the high number density is centred around *d* = 20–40 m and shape factor = 1.22 (figure 9*a*). This suggests that intensive lateral melt in the MEDEA image is in favour of generating small and rounded floes. On the other hand, for the WV3 image, the high number density is concentrated at the floe size of approximately 8–10 m, but spreads over a wide range of the shape factors (1.2–1.8) (figure 9*b*). Herman *et al*. [30] (laboratory experiment) reported the wave-fractured floes had an elongated shape with the eccentricity peak at approximately 0.8 (which is approx. 1.67 in the shape factor). This supports that a wide range of shape factors observed in the WV3 image is closely related to wave fracture in that area.

### (c) Concluding remark

**Figure 10.**
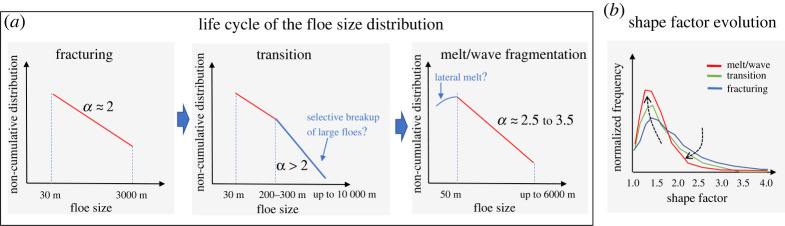
Conceptional illustration of (*a*) the life cycle of the floe size distribution and (*b*) shape factor evolution. (Online version in colour.)

In figure 10, we provide a conceptional summary of the life cycle of the floe size distribution proposed in this study. The initial floe size distribution of discrete floes is formed by the spring breakup and characterized by the single power-law behaviour with *α* ≈ 2. This power-law behaviour exists over the floe size range of *d* = ∼30–3000 m. The shape factor at this stage shows the peaks of the distributions occur at 1.4–1.5, indicating elongated floe shapes for the initial floe size distribution. During the transition stage, the same power-law behaviour (*α* ≈ 2) from the spring breakup is likely maintained in the floe size range smaller than 200–300 m, but large floes deviate from the initial state (i.e. downward curvatures) and form another power-law behaviour with a steeper slope (*α* = 2.54–3.46) over the floe size range of *d* = ∼300 to 3000 m. This suggests that the floe fragmentation may occur preferentially in large floes (*d* > ∼300 m), as large floes are more susceptible to break due to higher length-to-thickness ratios. Dynamic ice conditions such as strong wind/current or a low ice concentration likely enhance this preferential breakup through floe-floe interaction or deforming failure. At the final stage, the melt/wave fragmentation becomes the dominant process, and the floe size distribution returns to the single power-law behaviour but with steeper exponents (*α* = 2.5 to 3.5) than the ones from the fracturing cases. The single power-law behaviour exists over the floe size range of *d* = 70–2000 m for early melt and 70–1000 m for late melt.

The scaling effects were examined by comparing the floe characteristics derived from images with different image resolutions. For the transition cases, the floe size distribution derived from two TX images shows the power law regime over the floe size *d* = ∼300–400 to 3500–10 000 m with *α* = 2.44–2.66. For the early and late melt cases, more direct comparisons were possible as the TX SM images were available within 2 days of the MEDEA images at the same location. In this case, the *α* values derived from the TX SM images over the floe size *d* = 200–300 to 2000–6000 m were only slightly higher (approx. 0.3) than those from the corresponding MEDEA images. Collectively these results suggest that the power-law regime may be extended up to 10 000 m for the transition cases and up to 6000 m for melt cases, yet more studies are required to confirm the robustness of this scaling behaviour.

We identified three melt fragmentation factors: (a) deepened melt ponds along the ridges, (b) disintegration by heavily melted melt ponds and (c) breakage of thin ice. Our observations on melt fragmentation are limited to a single case during the MIZ experiment in the Beaufort Sea, so it is not certain whether melt fragmentation is widespread across the Arctic or is confined to the thermodynamically active western Arctic [60]. It requires further investigation. The effects of waves on the floe size distribution are not conclusive in our study. The Bering Sea example provides us with a clue of how the floe size distribution may look like under wave fracture before the melt, as its floe size distribution and shape factor are remarkably similar to the ones shown in the laboratory wave experiment [30].

Our examination of multi-satellite data provides some insights into the proposed idea of the life cycle of the floe size distribution of the Arctic sea ice, yet many questions remain to answer. Finally, in terms of addressing the hypothesis, we argue that the seasonal life cycle of the floe size distribution can be conceptualized in three stages (fracturing, transition and melt/wave fragmentation) and each stage has its own floe size distribution characteristics across different scales. Our study suggests the conceptional framework could be a good starting point in harmonizing existing datasets and identifying lacking observations (such as wave-fractured floes) to better inform the modelling development and improvement.

## Data Availability

MEDEA images are openly accessible from the GFL website (http://gfl.usgs.gov/). TerraSAR-X and WorldView-3 images cannot be shared due to the license agreement, but all other data can be obtained from UK Polar Data Centre (DOI: https://doi.org/10.5285/7dc6e19d-79fa-41f1-99a7-da408592382f - Multi-satellite floe size distribution of Arctic sea ice 2000–2020) [61]. The datasets supporting this article are provided in electronic supplementary material [62].
